# Predictive validity of the Lacey Assessment of Preterm Infants for motor outcome at 2 years corrected age

**DOI:** 10.1016/j.earlhumdev.2021.105334

**Published:** 2021-04

**Authors:** Anna M. Lukens, Naomi R. Winfield, Charlotte A. Xanthidis, Tomoki Arichi

**Affiliations:** a Children's Neurosciences, Evelina London Children's Hospital, Guys' and St Thomas' NHS Trust, London, UK; b Centre for the Developing Brain, School of Biomedical Engineering and Imaging Sciences, King's College London, London, UK; c University College London, London, UK; d Department of Bioengineering, Imperial College London, London, UK

**Keywords:** Preterm, LAPI, Lacey, Motor outcome, Predictive

## Abstract

**Introduction:**

The Lacey Assessment of Preterm Infants (LAPI) is a clinical tool used to assess neuromotor development in preterm infants at high risk of developmental problems. The aim of this study was to determine its predictive validity for estimating later motor outcome at 2 years of age, to ensure appropriate referral to early intervention and thus optimise the infant's outcome.

**Method:**

LAPI outcomes (usual or monitor) for preterm infants born between January 2012–2017 at a single tertiary level neonatal intensive care unit in London, UK were retrospectively reviewed. Predictive validity for later “moderate/severe” motor delay was determined by comparing LAPI outcomes with locomotor scores estimated using the Griffiths Mental Development Scales-Extended Revised (GMDS-ER) or Griffiths III at 2 years corrected age.

**Results:**

118 infants were included (GMDS-ER = 87, Griffiths III = 31). Infants classified as usual on the LAPI showed significantly less motor delay on the GMDS-ER locomotor subset at 2 years, compared to infants in the monitor group (usual = 2.00 months, monitor = 6.00 months; *p* = 0.001). Sensitivity was found to be only 47.37%, with higher specificity of 84.85%.

**Conclusion:**

The LAPI shows high specificity but low sensitivity for prediction of later motor delay. It may therefore be useful for screening lower-risk infants, however on-going monitoring would be advised. Further studies investigating the reliability of the LAPI and use in conjunction with other predictive tools to improve sensitivity are recommended.

## Highlights

• The LAPI demonstrates high specificity (85%) for prediction of 2-year motor outcome.
• A ‘usual’ LAPI outcome is strongly predictive of later normal motor outcome.
• However, the LAPI demonstrated lower sensitivity (47%).
• The LAPI may be a useful tool for screening lower-risk infants.
• Ongoing surveillance and monitoring for at-risk infants is strongly advised.

## 1 Introduction

Although survival rates of preterm infants have been steadily improving with advances in neonatal medicine, infants born extremely or very preterm (<32 weeks gestation at birth) remain at significantly increased risk of neurodevelopmental difficulties [1]. Early identification of neuromotor disorders such as cerebral palsy (CP) is therefore essential in the management of these vulnerable infants, as emerging research suggests early intervention can positively influence neuroplasticity and later motor outcomes [2]. It also ensures appropriate allocation of resources and access to early psychological support for parents.

Research shows that the use of appropriate assessment tools such as Prechtl's Assessment of General Movements (GMs) and Hammersmith Neonatal Neurological Examination (HNNE) can predict later CP with high sensitivity at term equivalent age or a few months of age [2,3]. However sensitivity and use during the preterm period itself is decreased or less evidenced [4] and many studies to date have investigated low risk or term infants [[5], [6], [7]]. This is important, as advances in neonatal care and outreach support have led to many preterm infants leaving hospital before term age, as early as 33 weeks postmenstrual age (PMA) [8]. It is therefore vital to have a neurological assessment tool valid at this age to plan for necessary ongoing community support.

The Lacey Assessment of Preterm Infants (LAPI) was designed to assess longitudinal motor development and neurological status specifically for preterm infants on the neonatal unit, to predict later motor outcome by classifiying infants as ‘usual’ or ‘monitor’ based on the findings [9].

Sensitivity for the LAPI for prediction of later CP has been reported as 75% to 86% when assessed beyond 33 weeks PMA [9,10]. This was lower when assessed at less than 33 weeks PMA (57%), possibly due to lower muscle tone at earlier ages potentially masking the presence of abnormal postures [9]. Furthermore, the high sensitivity reported in Lacey's original work used an older version of the LAPI, which used different classifications of ‘suspect’ and ‘abnormal’ findings. When determining predictive validity for ‘abnormal’ infants alone, sensitivity was markedly lower at 52% [9]. However, a more recent pilot study by Marcroft et al. [10] using the current version of the LAPI found an improved sensitivity of 75%. Specificity across studies is consistently high (83–98%), suggesting that the LAPI assessment could be used to identify infants at low risk of developmental difficulties to provide reassurance to families and help guide appropriate provision of early intervention resources [[9], [10], [11]].

Taken together, previous evidence suggests good predictive validity of the LAPI, making it a good candidate as a predictive assessment tool for later neuromotor outcome in the preterm period. However, most existing data used an older version which has been superseded and was tested on cohorts that pre-date changes in preterm care. Moreover, the majority of studies only compared LAPI scores with a binary outcome of later CP diagnosis. Comparison with a motor score or an extended classification including minor or major motor dysfunction may be clinically more useful, as a diagnosis of CP encompasses a wide spectrum of motor difficulties [12]. The aim of this study was therefore to determine the predictive validity of the LAPI when used to assess preterm infants before discharge home, to predict motor outcome on formal neurodevelopmental assessment at 2 years of age.

## 2 Method

A retrospective case note review was conducted at the Evelina London Children's Hospital (ELCH). Infants delivered between 2012 and 2017 at less than 32 weeks GA or weighing less than 1.5 kg at birth were eligible if they had at least one complete LAPI assessment on the neonatal unit and attended a follow-up appointment within the Evelina community services, between the ages of 18–30 months corrected age. Infants with a suspected or diagnosed congenital abnormality were also included, with the potential confounding effect later assessed using multiple linear regression. Local NHS Trust approval for the project was granted as a service evaluation.

The ELCH neonatal unit is a level 3 tertiary intensive care unit with 52 cots and is a regional referral centre for many neonatal specialties including cardiology, neurology and surgery across South-East England. All preterm infants that met the criteria were routinely assessed using the LAPI by a specialist neonatal or paediatric physiotherapist. The specialist neonatal physiotherapist was trained on the formal LAPI course, while all other physiotherapists were trained in-house by this physiotherapist.

All LAPI assessments were performed in accordance with the LAPI course manual on the neonatal unit when the infant was medically stable, off mechanical ventilation and able to tolerate handling. Assessments were repeated at weekly or fortnightly intervals as deemed appropriate until discharge. LAPI classification at the final assessment was used to determine ongoing therapy input. Infants classified as monitor were referred to community therapy services, to maximise the opportunity for early intervention [13].

Infants had a detailed neurodevelopmental assessment at 24 months, completed by a trained Paediatrician or Neonatologist using the Griffiths Mental Development Scale – Extended Revised (GMDS-ER) or Griffiths III. The Griffiths Scales of Child Development is a tool that assesses global development from birth to 6–8 years of age, providing an overall norm-referenced measure of child development, and profile of abilities across five domains; performance, language, co-ordination, personal-social and gross motor skills [14,15]. The GMDS-ER was used within the ELCH developmental clinic until 2016, when it was updated to the Griffiths III. The Griffiths Scales' locomotor subscale has been previously shown to accurately screen for motor disabilities and correlate with medical diagnosis [16,17]. The locomotor subscale has shown a moderate correlation of 0.66 (*p* < 0.05) between the GMDS-ER and Griffiths III, at a later age of 3–6 years [18]. For the purposes of this study, the GMDS-ER and Griffiths III groups were therefore analysed separately.

LAPI data (number of complete LAPI assessments, age at assessment, LAPI classification, and presence of atypical features) was collected from paper records for infants assessed between 1st January 2012 and 1st January 2017, that met the aforementioned inclusion criteria. For infants that had multiple complete LAPI assessments, the final LAPI classification was used for analysis, as this determined suitability for onward referrals [9]. Demographic information including GA, BW, and the presence of co-morbidities was also collected.

Data from the two-year neurodevelopmental assessment was collected from the UK national neonatal medical records application (BadgerNet,Clevermed Ltd., Edinburgh UK) and cross-referenced with outpatient clinic letters and community electronic records. Age-equivalent scores for each subset were recorded when available, otherwise raw scores were converted. A known diagnosis of CP or neurological difficulties was also recorded.

Data was analysed using Statistical Package for the Social Sciences (SPSS) software (IBM Corp. Released 2017, IBM SPSS Statistics for Windows, Version 25.0, Armonk NY, USA). A Mann-Whitney *U* test was used to analyse the data between the LAPI binary outcome, and locomotor age equivalent score or number of months delay. Multiple linear regression analysis was performed to establish coefficients for potential confounding factors that differed between groups and may influence outcome, such as GA, BW, and the presence of CLD, NEC and congenital abnormalities (with the latter three treated as dichotomous variables).

To determine predictive validity, infants were classified according to NNAP standards into categories of delay: no delay (0–3 months), mild (3–6 months), moderate (6–12 months) or severe (12+ months). Sensitivity, specificity, positive predictive value (PPV) and negative predictive value (NPV) were calculated using a 2 × 2 contingency table for prediction of “no/mild delay”, or “moderate/severe delay”. A Fisher's exact test was used to test statistical significance.

## 3 Results

**Fig. 1:**
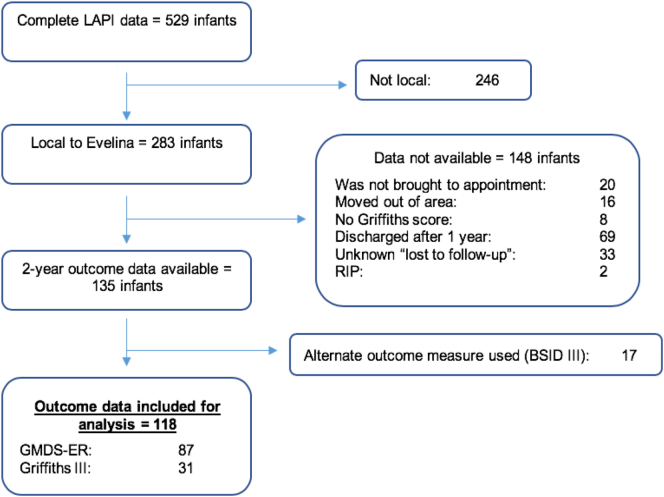
Flow diagram for study population. Key: BSID III = Bayleys Scales of Infant Development III, GMDS-ER = Griffiths Mental Development Scales – Extended Revised.

Complete LAPI data was available for 529 infants, of which only 118 infants had complete outcome data, therefore were included in the study (Fig. 1). Eighty seven infants were assessed using the GMDS-ER and 31 using the Griffiths III.

At the infant's final LAPI assessment, 94 (79.7%) were classified as usual and 24 (20.3%) as monitor, with a mean PMA of 36.03 weeks (SD 2.45). The median number of LAPI assessments completed per infant was 2 (range 1–4). The mean age of assessment for infants in the GMDS-ER and Griffiths III was 24.01 (SD 1.74) and 24.23 months (SD 0.87) respectively. Two tailed *t*-tests identified that infants in the GMDS-ER group had lower mean GA at birth (27.41 versus 28.87; *p* = 0.001), mean BW (956.9 g versus 1152.3 g; *p* = 0.002) and higher locomotor delay (2.50 versus 0, *p* = 0.03), therefore groups were kept separate for further statistical analysis (Table 1).

**Table 1:** Comparison of patient demographics between usual and monitor LAPI classification groups.

	Characteristic	Usual	Monitor	*P*-value⁎
GMDS-ER	N=	73 (83.9%)	14 (16.1%)	
	Gender: Male	32 (43.8%)	9 (64.3%)	0.24
	Female	41 (56.2%)	5 (35.7%)	
	Mean GA (weeks)	27.72 (2.07)	25.79 (2.19)	**0.002**⁎⁎
	Range GA (weeks)	23.71 to 33.57	23.57 to 29.42	
	Mean BW (g)	990.7 (289.0)	780.7 (264.3)	**0.014**⁎⁎
	Range BW (g)	560 to 1730	440 to 1400	
	Presence of (% of total):			
	CLD	40 (54.8%)	13 (92.9%)	**0.007**⁎⁎
	NEC	5 (6.8%)	4 (28.6%)	**0.034**⁎⁎
	IUGR	13 (17.8%)	2 (14.3%)	1.00
	Sepsis	18 (24.7%)	5 (35.7%)	0.51
	Genetic	2 (2.7%)	1 (7.1%)	0.41
Griffiths III	N=	21 (67.7%)	10 (32.3%)	
	Gender: Male	13 (61.9%)	8 (80%)	0.42
	Female	8 (38.1%)	2 (20%)	
	Mean GA (weeks)	29.08 (1.74)	28.42 (1.48)	0.31
	Range GA (weeks)	24.85 to 31.14	26.14 to 31.28	
	Mean BW (g)	1153.3 (296.7)	1150.0 (342.6)	0.98
	Range BW (g)	710 to 1700	540 to 1740	
	Presence of (% of total):			
	CLD	14 (66.7%)	7 (70%)	1.00
	NEC	0 (0%)	1 (10%)	0.32
	IUGR	4 (19%)	2 (20%)	1.00
	Sepsis	3 (14.3%)	2 (20%)	1.00
	Genetic	0 (0%)	1 (10%)	0.32

Key: BW = birth weight, CLD = chronic lung disease, GA = gestational age, GMDS-ER = Griffiths Mental Development Scales – Extended Revised, IUGR = intrauterine growth restriction, NEC = necrotising enterocolitis, SD = standard deviation.

Where appropriate, results shown as mean (SD) due to normal distribution of data.

⁎ Fishers exact test used to compare categorical variables and two-tailed *t*-test used for continuous variables.

⁎⁎ Statistically significant data (p < 0.05).

Only three infants had a diagnosis of CP at 2 years. Two of these had bilateral abnormal lower limb muscle tone but scored appropriately on the locomotor subset with no or mild delay (1 and 5.5 months delay). One infant had unilateral CP, presenting with severe delay on the locomotor subset (13 months). All three infants had a monitor LAPI classification.

**Fig. 2:**
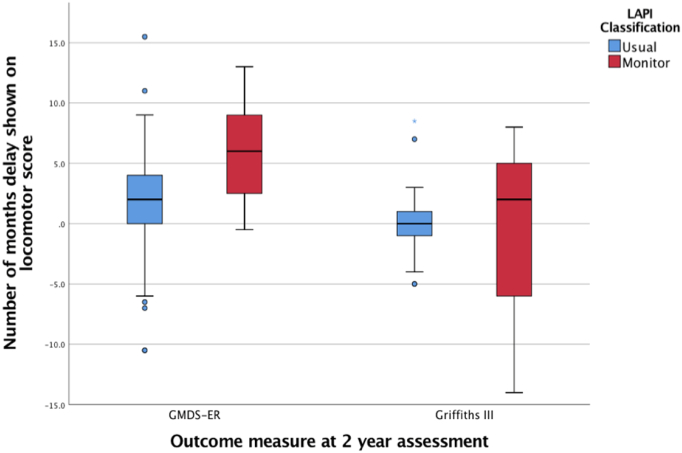
A boxplot to show locomotor delay between usual and monitor LAPI groups. Key: circles = outliers, asterix = extreme values, IQR = interquartile range. Results shown as median, IQR, minimum and maximum values.

A Mann-Whitney *U* test identified that infants classified by the LAPI as monitor showed significantly higher median motor delay (6.00 versus 2.00 months; *p* = 0.001) than infants classified as usual at their GMDS-ER 2-year assessment (Fig. 2). Within the Griffiths III group, although infants classified as monitor had higher motor delay (2.00 versus 0 months; *p* = 0.63), this was not statistically significant.

Multiple linear regression analysis was performed to take into account the following potential confounding variables: GA, BW, presence of CLD, NEC and genetic abnormalities. A ‘monitor’ LAPI outcome was found to be significantly associated with locomotor delay assessed by the GMDS-ER (*p* = 0.009), with a coefficient of 3.58 (95% CI 0.92 to 6.25). No other variables were found to be significant.

Within the GMDS-ER group, 65/73 infants classified as usual (89%) presented with no/mild delay, and 8 (11%) moderate/severe delay (Table 2). Of the infants classified as monitor, 7 (50%) demonstrated no/mild delay, and 7 (50%) moderate/severe delay.

**Table 2:** LAPI classification compared to no/mild or moderate/severe motor delay.

	Outcome	Usual N (%)	Monitor N (%)	P-value⁎
GMDS-ER	No/mild delay	65 (89.0%)	7 (50.0%)	**0.002**⁎⁎
	Moderate/severe delay	8 (11.0%)	7 (50.0%)	
Griffiths III	No/mild delay	19 (90.5%)	8 (80%)	0.58
	Moderate/severe delay	2 (9.5%)	2 (20%)	

Key: GMDS-ER = Griffiths Mental Development Scales – Extended Revised, N = number.

⁎ Fishers exact test used to compare categorical data.

⁎⁎ Statistically significant data (p < 0.05).

Of the infants assessed with the Griffiths III, similarly 19/21 (90.5%) classified as usual presented with no/mild delay and 2 (9.5%) with moderate/severe delay. Eight (80%) infants classified as monitor on the LAPI were classified as having later no/mild delay, while only 2 (20%) demonstrated later moderate/severe delay. Fishers exact test found a significant difference in outcome assessed with GMDS-ER, between infants classified as usual or monitor (*p* = 0.002). No significant difference was found between infants assessed with the Griffiths III.

Sensitivity and PPV for prediction of moderate/severe delay was low, 47.37 (95% CI 24.45 to 71.14) and 37.50 (95% CI 23.58 to 53.84) respectively, indicating that there is less than a 50% likelihood that the infant will show locomotor delay on the GMDS-ER, if classified as monitor (Table 3). Specificity and NPV were higher, 84.85 (95% CI 76.24 to 91.26) and 89.36 (95% CI 84.47 to 92.84), therefore if an infant presents as usual, they are highly likely to have a good motor outcome.

**Table 3:** Sensitivity, specificity, PPV and NPV of LAPI in predicting no/mild or moderate/severe motor delay.

	Sensitivity % (95% CI⁎)	Specificity % (95% CI⁎)	PPV % (95% CI⁎⁎)	NPV % (95% CI⁎⁎)
GMDS-ER	**46.67** (21.27 to 73.41)	**90.28** (80.99 to 96.00)	**50.00** (29.16 to 70.84)	**89.04** (83.42 to 92.92)
Griffiths-III	**50.00** (6.76 to 93.24)	**70.37** (49.82 to 86.25)	**20.00** (7.41 to 43.86)	**90.48** (77.58 to 96.31)
Combined	**47.37** (24.45 to 71.14)	**84.85** (76.24 to 91.26)	**37.50** (23.58 to 53.84)	**89.36** (84.47 to 92.84)

Key: CI = confidence intervals, GMDS-ER = Griffiths Mental Development Scales – Extended Revised, NPV = negative predictive value, PPV = positive predictive value.

⁎ Exact Clopper-Pearson CI.

⁎⁎ Standard logit CI.

## 4 Discussion

This is the first study to investigate the relationship between the current version of the LAPI with locomotor outcome at 2 years of age. Our findings suggest that there is a significant relationship between the LAPI classification and the locomotor subset of the Griffiths neurodevelopmental assessment, with infants classified as ‘usual’ scoring significantly higher in comparison to those in the ‘monitor’ group. Furthermore, we found that the LAPI has a high specificity and NPV for prediction of moderate/severe motor delay, but a comparatively low sensitivity and PPV. This suggests that clinicians using the LAPI can be relatively confident that infants classified as usual will have a normal locomotor outcome, but that a significant proportion of infants will be falsely identified as having potential later adverse outcome.

GMDS-ER locomotor scores at 2 years were significantly higher for infants classified as usual than those classified as monitor (*p* = 0.003), demonstrating significantly less motor delay (2.00 months versus 6.00 months; *p* = 0.001). These results support previous findings, in which infants within the ‘suspect’ LAPI group showed significantly lower scores than the ‘usual’ group on the Touwen motor assessment at 6 years of age, for all motor subsets including balance, co-ordination and fine-motor skills (*p* < 0.05) [11]. Linear regression analysis found a predicted delay of 3.58 months for infants classified as monitor, after accounting for the effects of GA, BW, presence of NEC, CLD and genetic abnormalities.

Sensitivity was found to be lower than reported in previous literature, at 47.4% (95% CI 24.5% to 71.1%), due to the relatively low number of true positives. This means a high number of infants were referred for closer monitoring and early intervention unnecessarily, potentially causing higher levels of parental anxiety and inefficient allocation of resources. This result is significantly lower than the figure of 75% reported by Marcroft et al. [10], although direct comparison is challenging as that study aimed to predict CP diagnosis rather than motor performance, and the population of infants differed from this current study with a lower GA, BW and higher prevalence of CP. Lacey et al. [9] reported varying levels of sensitivity of 52–86% for prediction of CP for infants when assessed beyond 33 weeks PMA, depending on whether atypical features were transient or abnormal. Sensitivity was only 52% for infants classified as ‘abnormal’, supporting findings of the current study. The globally reported higher sensitivity of 86% was determined when ‘unusual’ or transient findings were also classified as abnormal. On the current LAPI assessment, transient features are considered usual if the infant is over 33 weeks PMA at assessment. This difference may highlight difficulties with the current scoring system as original research suggests transient features show higher sensitivity, but currently do not result in a final monitor classification.

We found a high specificity of 84.9% (95% CI 76.2% to 91.3%) and NPV value, supporting previous literature and suggesting a high proportion of infants classified as usual had later normal locomotor outcome, therefore parents had received appropriate reassurance in the neonatal period. Marcroft et al. [10] similarly reported a high specificity of 89% (95% CI 71% to 98%), with wider CI due to the smaller sample size. Very high specificity of 98% was reported by Lacey and Henderson-Smart [11] and Lacey et al. [9], when comparing with neuromotor outcomes at 3 and 6 years old, perhaps due to a more robust diagnosis of CP at this later age when transient abnormal motor signs present in the first few years have resolved.

The LAPI is quick and easy to use, putting minimal stress on the infant. Use of the LAPI in conjunction with another tool such as the Prechtl GM's assessment or HNNE may therefore be appropriate to consider to improve predictive validity. However these tools similarly have relatively low sensitivity in the preterm period [4], perhaps reflecting greater developmental variability at this juncture [19]. Preterm infants are more likely to demonstrate atypical development in comparison to term infants, showing reduced variability within their movement patterns and difficulties with sensorimotor processing [19,20].

LAPI assessment alongside neuroimaging may provide higher diagnostic value for early identification and therefore direction of appropriate resources. Cranial ultrasound scans are often routinely used and readily available at the bedside to provide prognostic information for high-risk infants [21]. High specificity has been reported (90–95%) when sequential scanning is used, however sensitivity remains relatively low at 70–76% [3,21]. Alternatively, MRI scans at term equivalent age have shown higher sensitivity for prediction of neurodevelopmental outcome for preterm infants (86–100%) as more detailed neuroanatomy can be delineated, in particular the presence of white matter changes which are likely to impact on neurodevelopment [3]. However, MRI scans are costly and less accessible, but are increasingly being utilised within neonatal units to provide further diagnostic information [22]. In addition, maternal anxiety levels have been shown to reduce after receiving information from neuroimaging, more so following MRI scans [23].

### 4.1 Limitations

The sample population was from a single tertiary centre and therefore further work is required to establish how generalisable our results are to other clinical populations and centres. However our population appeared to be representative of infants born very preterm, and included those with the full spectrum of pathologies associated with prematurity. Data collected for infants lost to follow-up showed that those with no outcome data (52% of our local population) had a higher mean GA (30.31 versus 27.80; *p* < 0.001) and mean BW (1.32 kg versus 1.01 kg; p < 0.001), as well as lower incidence of CLD, NEC and sepsis. This may represent a bias in the data towards infants with higher levels of delay within the sample as those where the parents or clinicians had no concerns may not have returned for a developmental assessment.

Only three infants (2.5%) had been diagnosed with CP by their 2-year assessment, which is markedly lower than the reported prevalence of 6.2–14.6% reported in large cohort studies of high-risk preterm infants [3,24]. However, the low prevalence of CP in our population may reflect a delay in the diagnosis of CP by the clinicians within the neonatal follow-up service as a much higher proportion were found to have moderate to severe delay (16%) on the Griffiths assessment.

Although infants within the Griffiths III monitor group had lower median locomotor scores, this difference was not significant (*p* = 0.63). This lack of statistical significance may be due to the wide range of scores and small sample size, however it should also be considered that the Griffiths III is relatively new and has limited research within the preterm infant population.

### 4.2 Implications and further work

Due to the low sensitivity reported for the LAPI in this study, further research investigating use of the LAPI in conjunction with alternate measures, such as MRI, or comparison with Prechtl GMs are recommended. Future studies should also determine inter-rater and intra-rater reliability of clinicians with varying levels of experience. Research suggests there are windows of heightened neuroplasticity when brain development is heavily influenced by environmental input, particularly in the motor and sensory systems during the first few months after birth [25]. Altering the infant's environment during these windows may therefore promote growth and development of the musculoskeletal and neuromotor system, which may lead to better outcomes [26,27]. Therefore the ability to accurately identify infants at high risk of later neuromotor abnormalities would enable early intervention to start on the neonatal unit with close monitoring to try to maximise later outcomes [13].

## 5 Conclusion

Results from this study have shown that the current version of the LAPI has low sensitivity but high specificity in determining later motor delay for high-risk preterm infants. A usual LAPI classification prior to discharge can therefore provide some early reassurance to parents that the infant is at low risk of showing motor concerns at 2 years, however ongoing regular surveillance is strongly encouraged.

## Ethical approval

Local NHS Trust approval for the project was granted as a service evaluation. No ethical approval was required.

## Declaration of competing interest

The authors have stated that they had no interests which might be perceived as posing a conflict or a bias.
